# The landscape of alternative splicing in HIV-1 infected CD4 T-cells

**DOI:** 10.1186/s12920-020-0680-7

**Published:** 2020-04-03

**Authors:** Seyoun Byun, Seonggyun Han, Yue Zheng, Vicente Planelles, Younghee Lee

**Affiliations:** 1 Department of Biomedical Informatics, University of Utah School of Medicine, Salt Lake City, UT USA; 2 Department of Pathology, University of Utah School of Medicine, Salt Lake City, UT USA; 3 Huntsman Cancer Institute, University of Utah School of Medicine, Salt Lake City, UT USA

**Keywords:** Alternative splicing, *CCNT1*, HIV-1, *CD46*, CD4 T-cell

## Abstract

**Background:**

Elucidating molecular mechanisms that are altered during HIV-1 infection may provide a better understanding of the HIV-1 life cycle and how it interacts with infected T-cells. One such mechanism is alternative splicing (AS), which has been studied for HIV-1 itself, but no systematic analysis has yet been performed on infected T-cells. We hypothesized that AS patterns in infected T-cells may illuminate the molecular mechanisms underlying HIV-1 infection and identify candidate molecular markers for specifically targeting infected T-cells.

**Methods:**

We downloaded previously published raw RNA-seq data obtained from HIV-1 infected and non-infected T-cells. We estimated percent spliced in (PSI) levels for each AS exon, then identified differential AS events in the infected cells (FDR < 0.05, PSI difference > 0.1). We performed functional gene set enrichment analysis on the genes with differentially expressed AS exons to identify their functional roles. In addition, we used RT-PCR to validate differential alternative splicing events in cyclin T1 (*CCNT1*) as a case study.

**Results:**

We identified 427 candidate genes with differentially expressed AS exons in infected T-cells, including 20 genes related to cell surface, 35 to kinases, and 121 to immune-related genes. In addition, protein-protein interaction analysis identified six essential subnetworks related to the viral life cycle, including Transcriptional regulation by TP53, Class I MHC mediated antigen, G2/M transition, and late phase of HIV life cycle. *CCNT1* exon 7 was more frequently skipped in infected T-cells, leading to loss of the key Cyclin_N motif and affecting HIV-1 transcriptional elongation.

**Conclusions:**

Our findings may provide new insight into systemic host AS regulation under HIV-1 infection and may provide useful initial candidates for the discovery of new markers for specifically targeting infected T-cells.

## Background

Current treatments for HIV patients (e.g. highly active antiretroviral therapy, HAART) mainly aim to effectively suppress viral load, recover immunologic function, and prevent HIV from acquiring drug-resistant mutations [1–3]. Such treatments rely on preventing the infection from progressing to active AIDS, thereby improving survival. The complete elimination of the infection would be ideal, but is complicated by virus harbored in T-cells (i.e. provirus) [4]. Since the virus relies on the molecular machinery of the host T-cell for its replicative cycle during infection [4–8], understanding biological host factors involved in the viral life cycle may provide us with a basis for future drug development.

Genome-wide investigation of molecular changes in infected T-cells is a promising approach by which to identify a better strategy for curing HIV patients [9]. For example, genome-wide gene expression and DNA methylation profiling have successfully demonstrated changes in the molecular patterns of HIV-infected cells [9–12]. One such change occurs for nuclear factor IX (*NFIX*), which showed lower histone methylation in T-cells infected with HIV-1, consequently leading to its increased expression [11]. This increased *NFIX* may interact with the core-negative regulatory element (NRE) in the HIV-1 long terminal repeat (LTR), thus inhibiting the transcription of HIV-1.

Another potential mechanism by which HIV may regulate the molecular machinery of infected T-cells is alternative splicing (AS). Various immune cells use AS to regulate their proper activation or inactivation [13, 14]. Over 60% of genes produce immune cell-specific (i.e. T- or B-cell) AS isoforms [15, 16], and many such genes have been experimentally validated as relevant to immune responses, including cell surface receptors, cytokine-related genes, transcription regulators, RNA processing genes, intracellular signaling/transport genes, ion channels, and cytoskeletal genes [17]. For example, Fas cell surface death receptor (*FAS*) produces transcript isoforms with exon 6 skipping during peripheral blood mononuclear cell activation [18]. Another example is protein tyrosine phosphatase, receptor type C (*CD45*), in which skipping of exon 6 regulates immune signaling via protein kinase C (PKC) and Ras [17, 19]. A previous study carried out an exon array-based investigation of the transcriptome in uninfected and HIV-infected CD4 T-cells at 24 h, 48 h, and 72 h post-infection [4]. They reported that exon 54 of Inositol 1,4,5-Trisphosphate Receptor Type 1 (*ITPR1*), an ion channel that plays a role in lymphocyte activation, was skipped in HIV-1 infected T-cells. This suggests that a genome-wide scan of AS events in HIV-infected T-cells has the potential to identify additional molecular signatures of HIV-1 infection.

Here, we analyzed RNA-seq data from infected and non-infected T-cells, originally collected by B. Descours et al. [9]. We identified 427 genes that have differential splicing events in T-cells infected by HIV-1. These genes were significantly enriched for HIV replication-related pathways. Furthermore, as a case study, we experimentally validated an exon of cyclin T1 (*CCNT1)* that had increased skipping in infected T-cells; this and other splicing events could provide potential biomarkers for capturing infected T-cells. Our study provides not only additional insight into AS events as an underlying biological mechanism of HIV-1 infection, but also the opportunity to explore novel biomarkers for detecting infected T-cells.

## Methods

Additional file 1: Figure. S1 illustrates the overall pipeline of RNA-seq data analysis by which alternative splicing events were identified between infected and uninfected T-cells, along with functional interpretation of the alternative splicing events.

### Data and pre-processing

We obtained RNA-seq data (i.e. FASTQ files) from the NCBI Sequence Read Archive (SRA) (https://www.ncbi.nlm.nih.gov/sra, SRP id = SRP094482 and run ids = SRR5071107 - SRR5071122) [9]. The data was comprised of four sample groups: XH+, Vpx-treated infected T-cells indicating green fluorescent protein (GFP) (*n* = 4); XH-, Vpx-treated T-cells exposed to HIV, but HIV-negative T-cells (*n* = 4); X, Vpx-treated T-cells not exposed to HIV (*n* = 4); and NI, non-infected T-cells with neither Vpx treatment nor HIV exposure (*n* = 4) (Additional file 1: Figure S1, Step 1). The raw FASTQ file contained 75~100 million single-end reads of 101 nucleotides in length per sample. We performed general quality control, including evaluation of the reads quality and average GC content in each base, of whole reads using the FastQC package v0.11.4 [20] (Additional file 1: Figure S1, Step 2). We then removed contaminants that may have originated from adaptor or linker sequences using Trimmomatic v0.32 [21]. After this preprocessing, we mapped the reads to the human reference genome (GRCH37.75 based on hg19 reference sequence) using TopHat v2.1.1 [22]. All samples had unique mapping rates of approximately 80%. Among the multiple mapped reads in our significant 494 AS events, approximate 84% mapped to conserved regions. The remainder of multiple mapped reads account for only 0.66% of mapped reads that was used for determining the 494 AS events.

### Identification of AS events by RNA-seq

**Fig. 1:**
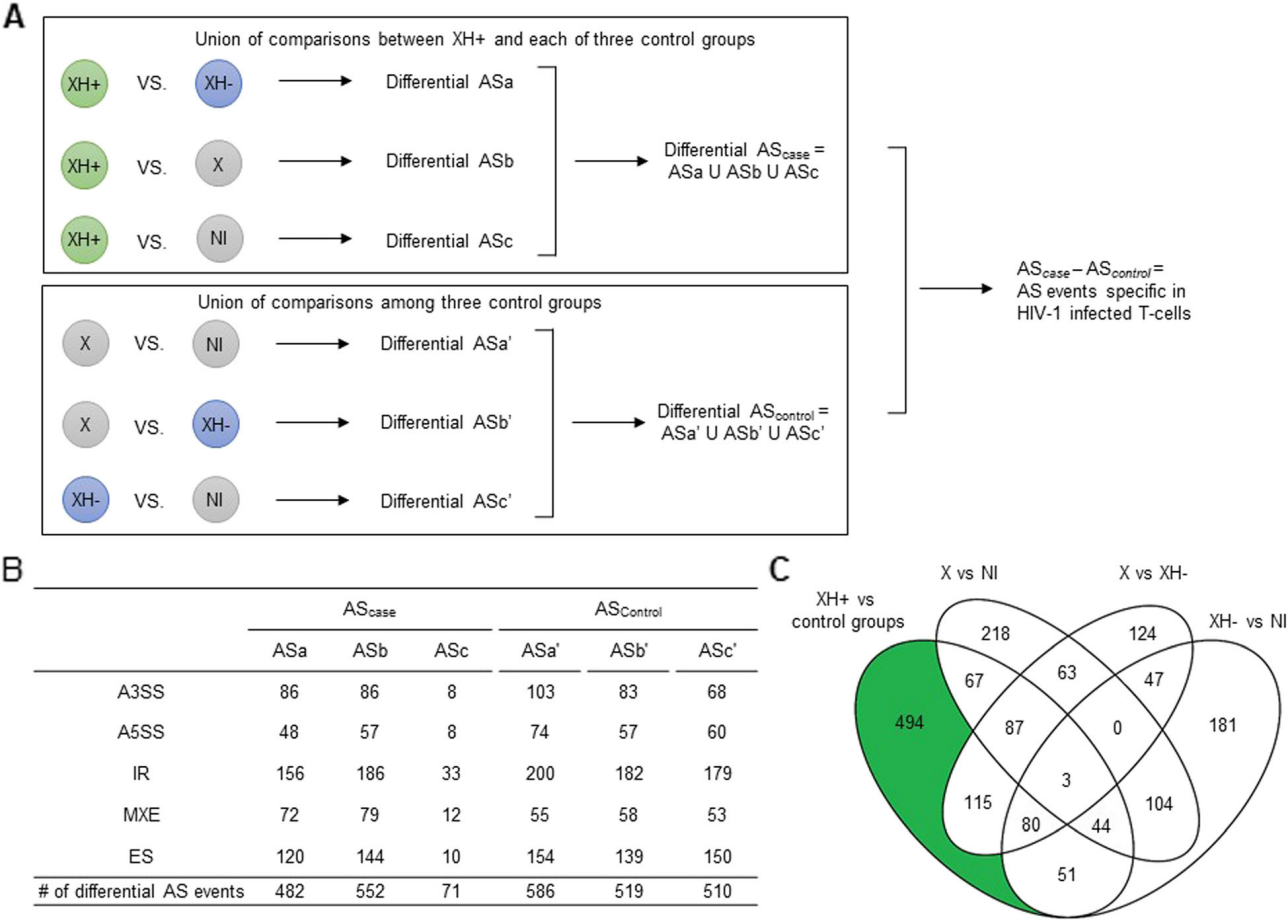
Identification of alternative splicing events in the HIV-infected T-cells. **a** Summary of the comparison process by which significant AS events were identified for HIV-infected cells. **b** Counts of differentially alternative spliced events observed in each group. A3SS, alternate 3′ SS; A5SS, alternate 5′SS; IR, intron retention; MXE, mutually exclusive exons; ES, exon skipping. **c** Venn diagram presenting the overlap of events between the four groups. There are 494 events unique to XH+ in 427 genes

We identified AS events in junction reads using rMATs v3.2.5 [23]. We considered all possible AS events: exon skipping (ES), alternative 5′ splicing site (A5SS), alternative 3′ splicing site (A3SS), intron retention (IR), and mutually exclusive exons (MXE). The rMATs was used to filter out multiple mapped reads, and reads spanning exon-exon junctions (junction reads) were used for identifying ES, A5SS, A3SS, and MXE. Also, for estimation of IR, rMATs used reads spanning exon-exon junctions and in retained introns. We estimated the expression level of each AS event, denoted as percent spliced in (PSI), the fraction of alternatively spliced exons estimated from exon-exon junction reads (Additional file 1: Figure S1, Step 3). A PSI value of zero means that all mRNAs in a given gene exclude the given exon, while a value of 1 indicates that the given exon is never skipped (i.e., a constitutive exon). We first compared the expression of each identified AS event between the case group of HIV-1 infected T-cells and each control group of non-infected T-cells (i.e., XH+ vs. XH-, XH+ vs. X, and XH+ vs.NI) and collected the union of differentially expressed AS events from each comparison. Next, To obtain a pure set of differentially expressed AS exons between case and controls, we performed additional comparisons between every possible pair of controls (i.e., XH- vs. X, XH- vs. NI, and X vs. NI). We then excluded differential AS exons that were also identified in these control group comparisons from the union of case events (Fig. 1a). An AS event was considered significant if it had FDR-corrected *p*-value < 0.05 and PSI > 0.1, i.e. > 10% difference in expression between groups.

### Gene set enrichment analysis

For the functional interpretation of identified AS genes, we conducted gene set enrichment analysis over canonical pathways and Gene Ontology (GO) terms using ConsensusPathDB v32 (CPDB; http://cpdb.molgen.mpg.de/) [24]. Pathways and terms were considered significant at FDR < 0.05. To reduce the redundancy of GO terms, we limited the GO hierarchy level from two to four categories and filtered out potentially false positive GO terms using GO-Module v1.0 [25]. In addition, for GO terms with similar functions, we then selected only the most significant term (Additional file 1: Figure S1, Step 4).

### Network analysis

We constructed a protein-protein interaction network for the set of identified AS genes using StringDB v10.5 [26] (Additional file 1: Figure S1, Step 4). We selected interaction relationships between genes with the highest confidence > 0.9 and without disconnected nodes in the network.

### Visualization of protein 3D structure

As a case study for the functional impact of AS, we investigated the functional impacts of AS events identified in two genes, *CCNT1* and the CD46 molecule (*CD46*), on their protein domains and 3D structures. To determine the alteration of CCNT1 protein structure, we obtained the X-ray crystallography structure of its canonical protein product (PDB id: 5l1z) from the Protein Data Bank [27]. Protein 3D structures were visualized with PyMol v1.3 [28] (Additional file 1: Figure S1, Step 4).

### Experimental validation of CCNT1

Cell culture: HEK293 cells were cultured in DMEM (Gibco, US) containing 10% FBS (Atlanta, US), 2 mM L-Glu (Gibco, US). Primary cells were isolated from peripheral blood from healthy donors, obtained by venipuncture. Primary cells were cultured in RPMI1640 (Gibco, US) containing 10% FBS, 2 mM L-Glu and 30 IU/ml IL-2 (Stem Cell Technologies, US). R^2^ = 0.03.

Virus production: Pseudotype viruses, pNL4.3-deltaEnv-nLuc-2ANef -VSVG, were produced by co-transfecting pNL4.3-deltaEnv-nLuc-2ANef (produced by Laura Martins in our lab) and pCMV-VSVG (Addgene) into HEK293 cells. After 2 days, cell supernatant was collected and filtered with a 0.22um filter. Viruses were titrated on SupT1 cells and stored at − 80 °C.

HIV latency assay: TCM cells were generated and cultured as previously described (Bosque A, Planelles V. Induction of HIV-1 latency and reactivation in primary memory CD4+ cell. Blood 2009, 113:58–65). Briefly, naïve cells from heathy donor were activated by anti-human IL-4 (Peprotech, US), anti-human IL-12 (Peprotech, US), TGF-β1 (Peprotech, US) and Dynabeads CD3/CD28 (Invitrogen, US) for 3 days. At Day 4, beads were removed and activated cells were cultured in fresh medium. At Day 7, cells were infected by pseudotyped pNL4.3-deltaEnv-nLuc-2ANef -VSVG. At Day 10, CD4 and P24 levels were checked by flow cytometry. CD4+ cells were isolated by a magnet kit (ThermoFisher Scientific, US). At Day 12, uninfected cells, CD4+ cells and CD4- were collected for qPCR test. At Day 20, uninfected cells and CD4+ cells were collected for qPCR test.

qPCR: RNA from cell samples were isolated according to the manual procedure of RNease Mini Kit (Qiagen, US). Reverse transcription was done using SSIV First Strand Synthesis System (Invitrogen, US). qPCR was done using Platinum SYBR Green qPCR SuperMix (Invitrogen, US).

Primers for CCNT1a: (Forward) AACAGCCTGCATTTGACCAC and (Reverse) ATCTCCCAATTGGACCACTTG. Primers for CCNT1b: (Forward) CAACCAACAGAACTGACACTG and (Reverse) ATCTGTTCCTCGGTCATCTG. Program on LightCycler480 (Roche, US): step 1: 50 °C 2 min. Step 2: 95 °C 2 min. Step 3: 45 cycles of 95 °C 30s, 56 °C 60s and 72 °C 30s.

## Results

As described in the Methods, we conducted multiple comparisons to identify the set of differential AS events particular to HIV-1 infected T-cells. We identified 482, 552, and 71 statistically significant AS events (FDR < 0.05 and PSI > 0.1) in three case-control comparisons, XH+ vs. XH-, XH+ vs. X, and XH+ vs. NI, respectively (see the Methods and Fig. 1a); these collectively comprised 941 AS events across 751 genes (Fig. 1b). After exclusion of 447 statistically significant AS events (379 genes) that were also detected in at least one of three control group comparisons (Fig. 1c), we were left with 494 AS events (427 genes). This set of 494 AS events (427 genes, including 20 cell surface-related, 35 kinase, and 121 immune-related genes) were considered to be uniquely differentially expressed in infected T-cells. The results are summarized in Additional file 2: Table S1.

### AS genes are enriched in pathways relevant to the HIV-1 life cycle

To gain insight into the molecular functions of the 427 AS genes and their roles in the HIV-1 life cycle, we performed gene set enrichment analysis for canonical pathways and Gene Ontology (GO) terms using CPDB (see the Methods). After reducing redundant terms, we identified 23 significantly enriched pathways (FDR < 0.05; Additional file 3: Table S2, Additional file 1: Figure S2). These included pathways related to the late phase of HIV-1 infection and transcriptional reactivation of HIV, such as “Late Phase of HIV Life Cycle,” “HIV Transcription Initiation,” “Formation of HIV-1 Elongation Complex Containing HIV-1 Tat,” “Transcriptional Regulation by TP53,” “Clathrin Derived Vesicle Budding,” and “Membrane Trafficking.” The “Late Phase of HIV Life Cycle” pathway encompasses processes spanning HIV-1 transcriptional activation through budding and maturation, including HIV-1 intracellular trafficking and assembly [29]. The TP53 pathway is well-known to negatively regulate HIV-1 transcription by inactivating its long terminal repeat (LTR) promoter. Membrane trafficking is also an important pathway for the recruitment and assembly of proteins required for HIV-1 budding. These results suggest that our 427 AS genes are implicated in HIV-1 replication in T-cells, and that there is a need to elucidate differential AS underlying host molecular mechanisms affecting HIV-1 genome replication (i.e. activation of HIV-1 transcription, assembly, and budding).

Within the set of 427 AS genes, 21 non-redundant GO terms were additionally overrepresented (FDR < 0.05), including “positive regulation of immune effector process” (GO:0002699), “regulation of cell cycle” (GO:0051726), and “IkappaB kinase activity” (GO:0008384) (Additional file 4: Table S3, Additional file 1: Figure S3). Enrichment in immune response-related GO terms was expected. Genes in these terms regulate cell cycle arrest and stimulate HIV-1 LTR-driven transcription [30, 31] in the infected T-cells. Meanwhile, IkappaB kinase is one of the key regulators of viral replication, in which an AS event may produce a novel inhibitor of HIV-1 replication [32]. Therefore, the enrichment analysis suggests a potential for AS to play a role in the HIV-1 infection within infected T-cells by modulating immune-related genes and pathways related to HIV-1 replication.

### Protein interaction network

**Fig. 2:**
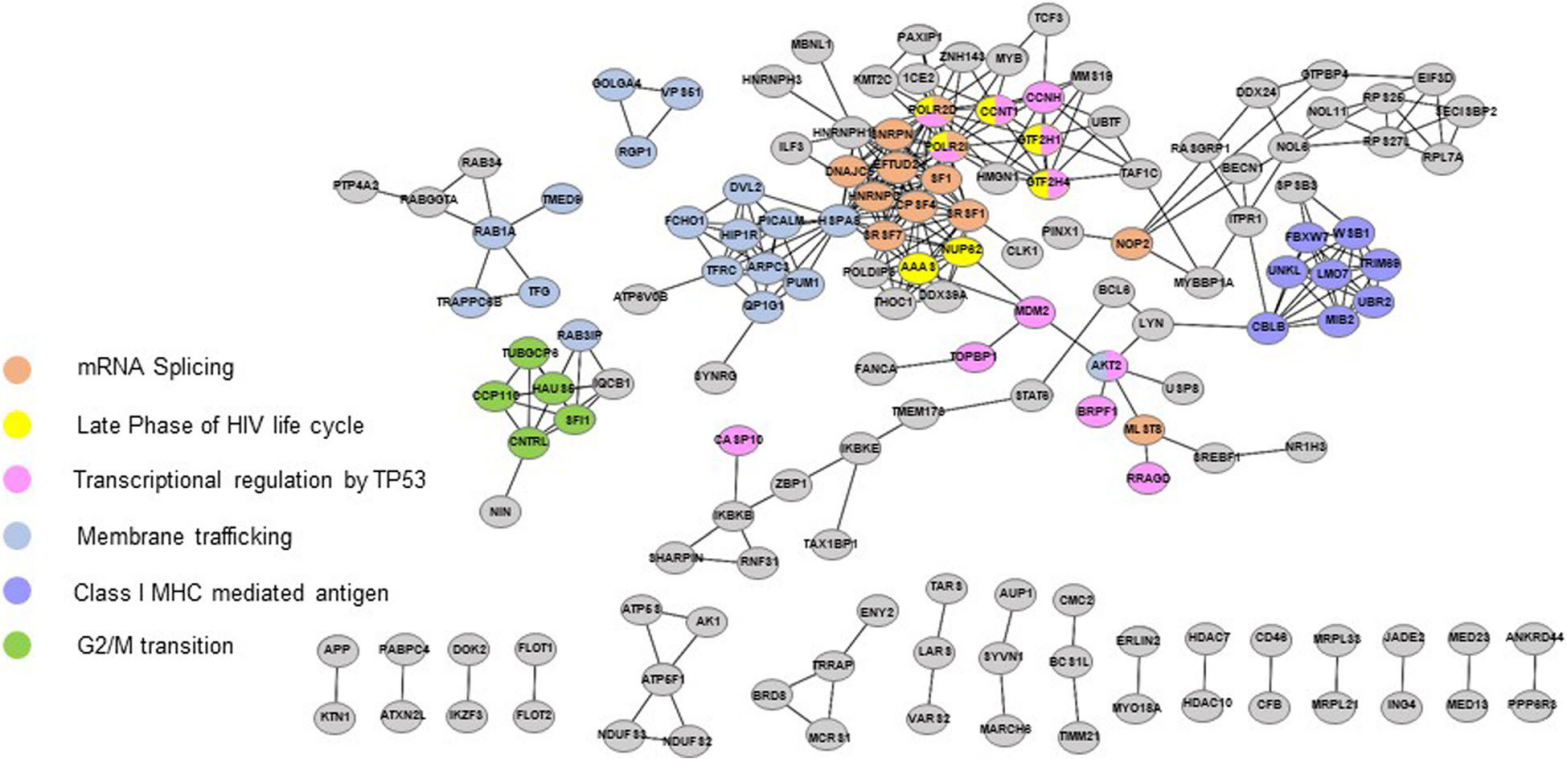
Functional analysis of 427 AS genes. Protein interaction network generated for differentially alternatively spliced genes (*N* = 427) using only interactions with highest confidence > 0.900 (*N* = 148). We found six key subnetworks involved in modulating in HIV-1 infection: Class I MHC mediated antigen, Regulation by TP53, Vesicle-mediated transport, mRNA splicing, Late phase of HIV life cycle, and G2/M transition

**Fig. 3:**
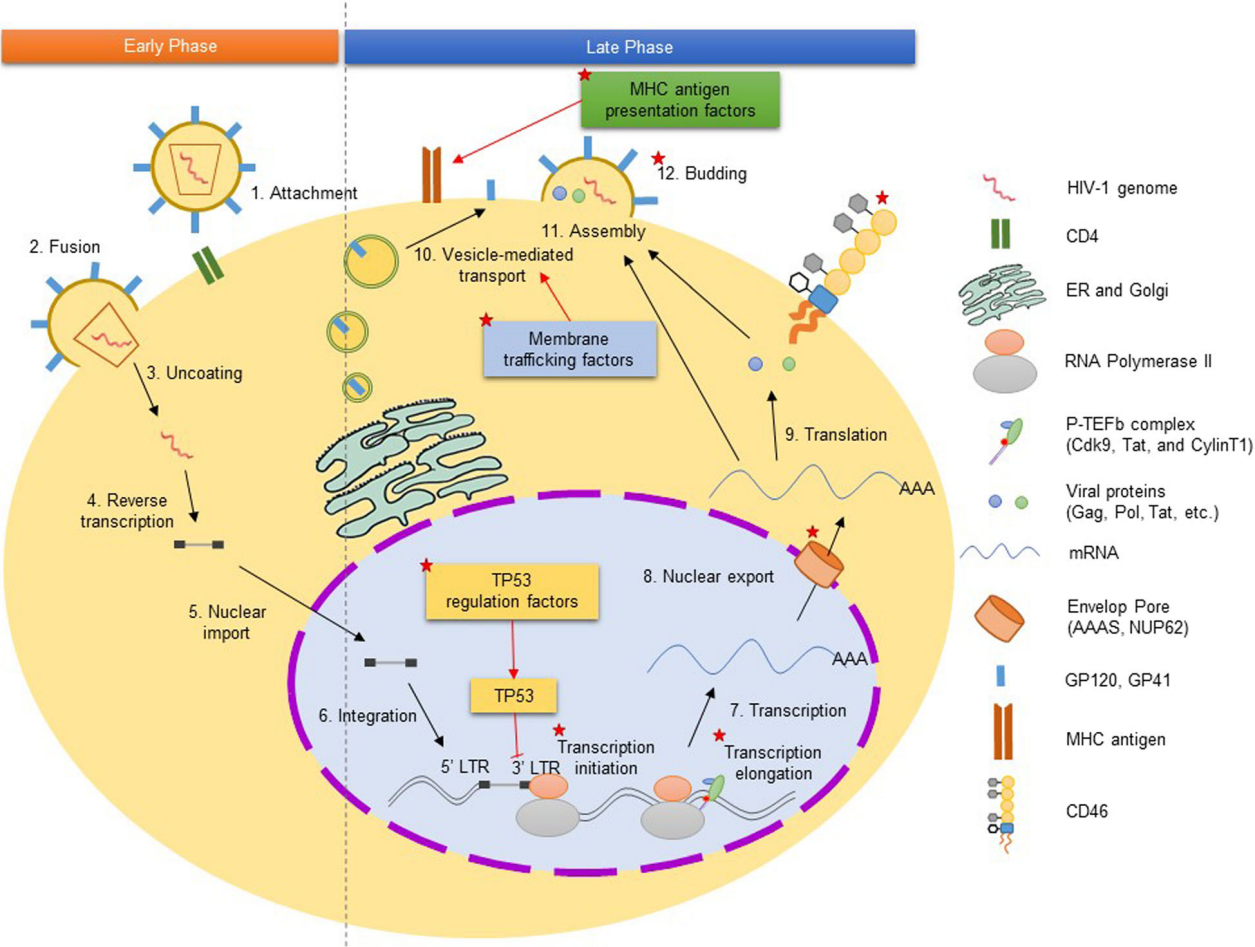
An implication of AS genes in HIV life cycle within CD4^+^ T-cells. There are twelve steps of the HIV virus replication cycle; the first five comprise the early phase, and the last seven the late phase. Highlighted boxes are significant protein interaction subnetworks from Fig. 2 (MHC antigen, TP53 regulation, membrane trafficking factors)

To gain further insight into the molecular signature of AS events related to HIV-1 infection, we constructed a protein-protein interaction network for the 427 identified AS genes. We then superimposed the significantly enriched canonical pathways and GO terms onto the network, identifying topological neighbors. At the network level, topological distance refers to the functional similarity of two proteins; topologically-neighboring genes are likely to have the same or similar functions [33]. In this analysis, our 427 AS genes functionally grouped into six subnetworks related to HIV-1 infection in T-cells, including regulation by TP53, vesicle-mediated transport, late phase of HIV life cycle, G2/M transition, and Class I MHC mediated antigen **(**Fig. 2). As mentioned above, TP53, vesicle-mediated transport, and late phase of HIV life cycle pathways all play functional roles in HIV-1 replication in T-cells; meanwhile, regulation of the G2/M transition pathway by HIV-1 viral infectivity factor induces G2 cell cycle arrest [34]. Class I MHC mediated antigen genes are also related to immune system function in infected T-cells. Finally, we created a schematic of the HIV-1 life cycle in T-cells that summarizes its overall modulation by AS (Fig. 3).

### Case study

As described above, our AS genes were enriched in pathways related to HIV-1 replication. In other words, the modulation of these genes by AS may be important for HIV-1 replication. As a case study, we selected a gene for functional analysis, *CCNT1*. We present further analysis and discussion of this gene’s potential role as a modulator of HIV-1 transcription and a marker specific to infected T-cells.

**Fig. 4:**
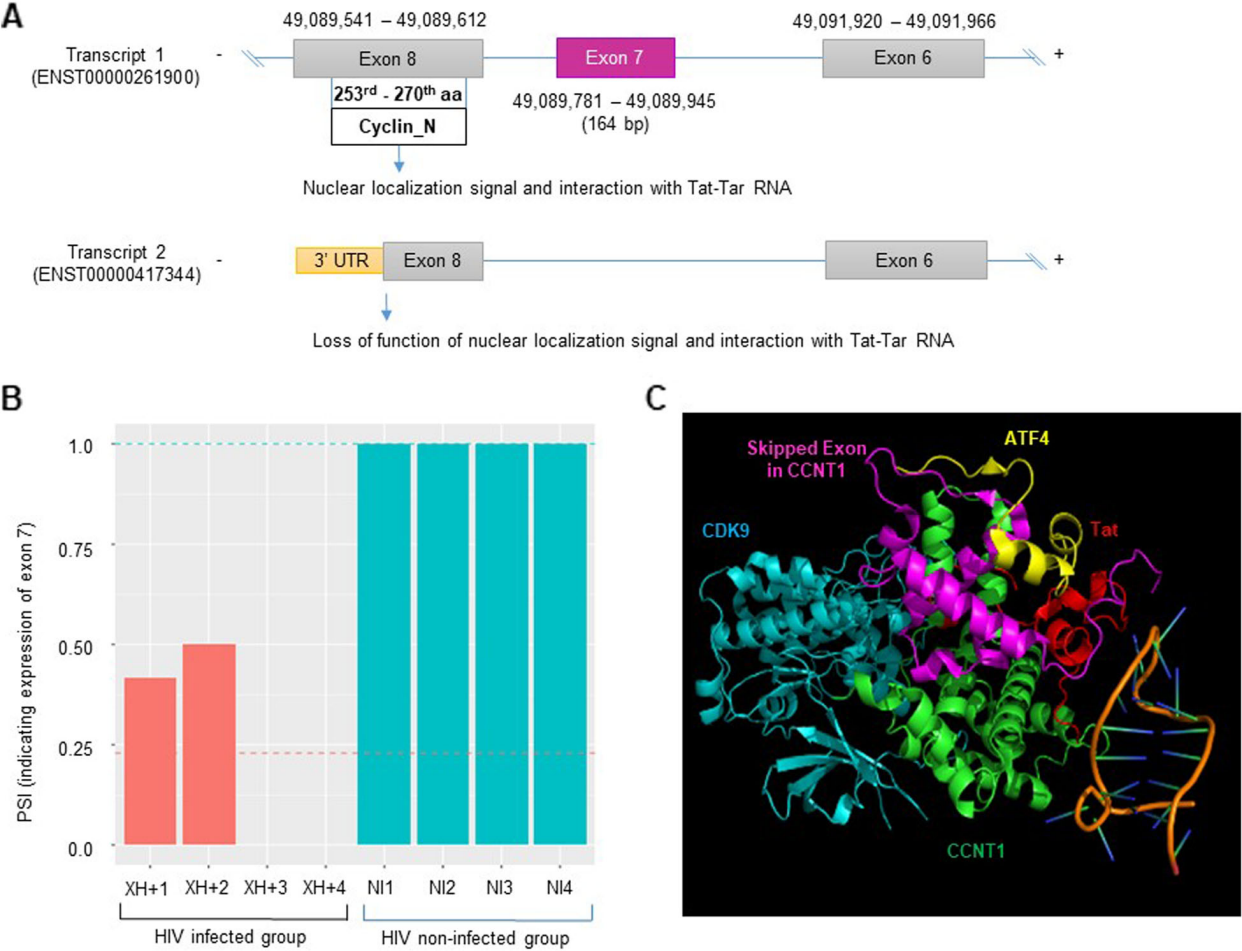
Skipping of *CCNT1* exon 7 in HIV-infected T-cells. **a** Effect of exon 7 skipping on *CCNT1* transcript and the Cyclin_N motif. **b** Reduced expression of exon 7 in XH+ samples compared to the three control groups. **c** X-ray crystallography of the P-TEFb complex (obtained from PDB). The region colored purple is encoded by the skipped exon

**Fig. 5:**
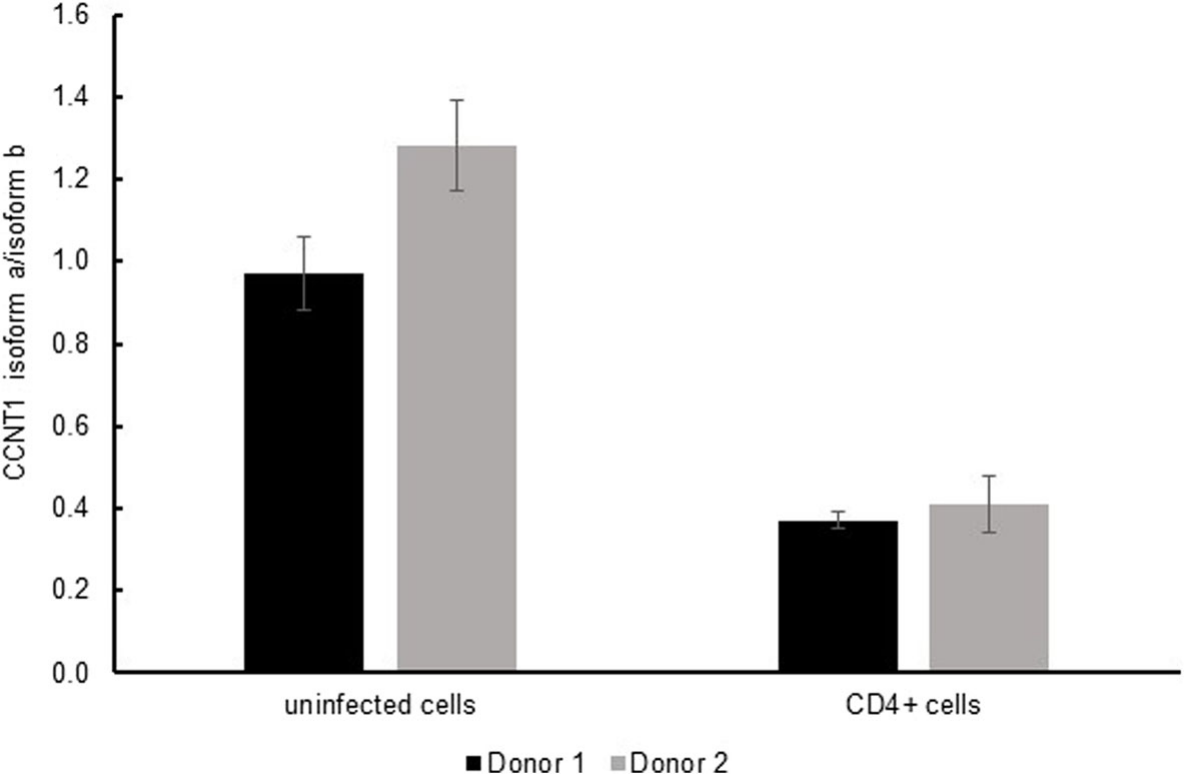
The ratio of gene expression of *CCNT1* isoform a and isoform b. Naïve CD4 cells from healthy donors were isolated and activated. Activated CD4 cells were infected by pseudo-typed HIV viruses for 2 days. At Day20 CD4+ cells containing latently infected cells and uninfected cells were isolated using a magnetic CD4+ isolation kit. Gene expression of *CCNT1* isoforms in uninfected cells and CD4+ cells was measured by qPCR. *CCNT1* isoform a/isoform b was calculated and shown

*CCNT1* is a member of the cyclin c subfamily and a component of the positive transcription elongation factor b (P-TEFb) complex with CDK9 and tat, making it essential for transcriptional elongation from the HIV-1 viral genome [35, 36]. In this study, we recapitulated a previously-identified exon 7 skipping event (164 base pairs) that may introduce an early stop codon, resulting in a potentially truncated protein or one subject to nonsense-mediated decay [37] (Fig. 4a). Exon 7 tends to be more skipped in HIV-1 infected T-cells than in non-infected (FDR = 3.10 × 10^− 10^) **(**Fig. 4b), which we validated experimentally (Fig. 5). As described in Fig. 4a, skipping of *CCNT1* exon 7 prevents it from encodes the Cyclin_N domain (PF00134); a protein that lacks this domain due to exon skipping may be unable to participate in the P-TEFb complex with Tat [38]. Figure 4c shows the 3D protein structure with the affected region highlighted in purple, adjacent to Tat. Furthermore, *CCNT1* function is known to be greatly regulated by alternative splicing [39, 40]. Therefore, this exon skipping event may be an important factor for inhibiting HIV-1 transcription through repression of the P-TEFb complex.

## Discussion

The paper in which the RNA-seq data analyzed in this study was originally published focused on genes differentially expressed between latently infected and non-infected T-cells. They identified overexpressed *CD32a* in the latently-infected XH+ group, suggesting that CD32a could be a potential latent-specific biomarker. However, there is an ongoing debate on whether this paper properly distinguished resting T-cells from activated CD4 T-cells [41]. In other words, expression of CD32a may not be solely enriched in resting T-cells [42, 43]. While there is an additional issue in the original study’s definition of the latently-infected XH+ group, this group can at least be confidently defined as infected because integration of the HIV-1 genome was confirmed by GFP [9]. Therefore, in this study we focus on infected CD4 T-cells.

Meanwhile, we experimentally validated that exon 7 of *CCNT1* was more frequently skipped in T-cells confirmed to be latently infected (Fig. 5 and Additional file 1: Figure S4) in our experimental system (see the Methods). This skipping event may lead to loss of *CCNT1* function (Cyclin_N) to interact with Tat (Fig. 4a) [44, 45]. Notably, CCNT1/CDK9 complex formation with Tat is crucial for activating transcription of the HIV-1 genome. CCNT1 is one of the P-TEFb complex components, and it plays an essential role in Tat-mediated HIV-1 transcription, activating the HIV-1 LTR promoter through direct interaction with Tat protein [44, 45]. In fact, there is a protein structure assay to identify how the complex specifically interacts with Tat [46]. HIV-1 transcriptional regulation is one of the most important factors in understanding the HIV-1 life cycle during infection, including latency. *CCNT1* is a candidate gene that may act as a key factor for controlling transcription of the HIV-1 genome, thus we selected this gene as a case study. Our experiment using RT-PCR suggests that the CCNT1/CDK9 complex is less abundant in latently infected CD4 T-cells, and enriched upon the reactivation of latent HIV-1 [47]. Therefore, our result suggests that exon 7 skipping of *CCNT1* may be functionally important for maintaining the latent phase of an HIV-1-infected CD4 T-cell as it leads to the loss of the key domain for P-TEFb complex constitution, ultimately contributing to the transcriptional inactivation of HIV-1.

To our knowledge, this study is the first of its kind, having been designed to comprehensively explore alternative splicing events in HIV-1-infected T-cells using RNA-seq. We used the rMATS package, which is especially well-designed for replication and group comparison of alternative splicing rates (i.e. PSI values). As observed outcomes may be specific to assembly tools, we conducted a cross-check of our results from rMATS with one of the most popular tools for estimating PSI value, MISO [48]. rMATS outcomes were highly matched with MISO outcomes; the correlation of PSI values between rMATS and MISO for all significant splicing events identified was 0.9 (median correlation). We showed that the 427 identified AS genes were enriched in functional relevance to HIV-1 replication and the HIV-1 infection, suggesting that HIV-1 may be implicated in regulating its host T-cell via alternative splicing. We constructed the hierarchically clustered heatmap to provide an expression trend of AS exons with PSI values across the cohorts, and XH+ (infected group) samples are clustered closely (Additional file 1: Figure S5). Furthermore, the set of AS genes included genes translated into cell surface proteins, which may provide potential biomarkers for detecting infected T-cells. As an example, *CD46* encodes a cell membrane protein that contains four short consensus repeats, a cytoplasmic domain, and a Ser, Thr, Pro-rich region (STP domain). This gene is known to have mutually exclusive exons (exon 7 and 8) (Additional file 1: Figure S6A), and we observed that exon 7, encoding the STP domain (Additional file 1: Figure S6B), was more likely to be skipped in infected T-cells (Additional file 1: Figure S6C), generating an aberrant protein product that lacks the STP region and may be useable as a marker.

A couple of limitations of the present study needs consideration. Although we presented experimental results from our case study splicing event in *CCNT1*, we did not validate other AS findings either in vitro or in an independent cohort. Another limitation is the small sample size (*n* = 4 each group). Therefore, further experimental validation or replication with independent data is required from future studies.

## Conclusion

Here, we present the potential of alternatively spliced genes as regulators and biomarkers in infected CD4 T-cells. We conclude that the study of alternative splicing may confer additional understanding of the molecular mechanisms underlying infection of T-cells as well as better treatment strategies for the early elimination of HIV-1.

## Supplementary information

**Additional file 1: Figure S1.** Overview of AS analysis in HIV-infected and non-infected human primary resting CD4+ T-cells. We obtained RNA-seq data for infected and non-infected cells from SRA (reference number SRR5071107-SRR5071122). There are four different treatment groups, and group contained four different cells. We then performed general quality control on the reads, aligned them to the hg19 human reference genome, and selected junction reads to identify alternatively spliced exons. Next, we identified mRNAs with differential alternatively splicing between the case group and each control. The percent spliced in (PSI) ratio indicates the level of differential exon expression. We adjusted q value < 0.05, and used PSI > 0.01 as the cutoff for differential splicing. Finally, we performed functional gene set enrichment analysis and classified genes with differential spliced exons in HIV infected T-cells according to their functional roles. **Figure S2.** 23 canonical pathways enriched in 427 HIV-associated AS genes. The red line indicates the cutoff FDR value, q < 0.05. **Figure S3.** 21 GO terms enriched in 427 HIV-associated AS genes. GO Biological Process (BP) and Molecular Function (MF) terms were considered significant at an FDR corrected q-value < 0.05 (red line). **Figure S4.** A plot for providing evidence to select quiescent cells. Ki67 is a proliferation marker, which is not expressed in quiescent cells but is expressed in dividing cells. The graph below shows the ki67 level of cells at Day19 (red line), which was negative, indicative of lack of cell division. **Figure S5.** A hierarchically clustered heat map with PSI levels of significant AS events. Groups are annotated by colors in the top of row, and types of AS exons are annotated by colors in the left column. **Figure S6.** Skipping of *CD46* exon 7 skipping in the HIV-infected T-cells. (A) Skipping of exon 7 in transcript 2 and retention of exon 8 (i.e. mutually exclusive exon event) affects the entire STP motif. (B) Location of the STP domain in *CD46* protein. (C) Reduced expression of exon 7 in HIV-infected samples. (D) Protein structure of *CD46* with the STP domain indicated in purple.

**Additional file 2: Table S1.** List of AS exons in the infected T-cells.

**Additional file 3: Table S2.** List of AS genes in the enriched canonical pathways.

**Additional file 4: Table S3.** List of AS genes in the enriched GO terms.

## Data Availability

RNA-seq data (i.e. FASTQ files) used in this study is available in the NCBI Sequence Read Archive (SRA): https://www.ncbi.nlm.nih.gov/sra, SRP id = SRP094482 and run ids = SRR5071107 - SRR5071122.

## Abbreviations

A3SS: Alternative 3′ splicing site
A5SS: Alternative 5′ splicing site
AS: Alternative splicing
ES: Exon skipping
FDR: False discovery rate
GFP: Green fluorescent protein
HIV-1: Human immunodeficiency virus 1
IR: Intron retention
MXE: Mutually exclusive exons
PSI: Percent spliced in
