# Supplementary material for: The landscape of alternative splicing in HIV-1 infected CD4 T-cells
Source: BMC Med Genomics. 2020 Apr 3;13(Suppl 5):38. doi: 10.1186/s12920-020-0680-7 (PMC7118826; doi:10.1186/s12920-020-0680-7)
Supplement: Supplementary file 1 — Additional file 1: Figure S1. Overview of AS analysis in HIV-infected and non-infected human primary resting CD4+ T-cells. We obtained RNA-seq data for infected and non-infected cells from SRA (reference number SRR5071107-SRR5071122). There are four different treatment groups, and group contained four different cells. We then performed general quality control on the reads, aligned them to the hg19 human reference genome, and selected junction reads to identify alternatively spliced exons. Next, we identified mRNAs with differential alternatively splicing between the case group and each control. The percent spliced in (PSI) ratio indicates the level of differential exon expression. We adjusted q value < 0.05, and used PSI > 0.01 as the cutoff for differential splicing. Finally, we performed functional gene set enrichment analysis and classified genes with differential spliced exons in HIV infected T-cells according to their functional roles. Figure S2. 23 canonical pathways enriched in 427 HIV-associated AS genes. The red line indicates the cutoff FDR value, q < 0.05. Figure S3. 21 GO terms enriched in 427 HIV-associated AS genes. GO Biological Process (BP) and Molecular Function (MF) terms were considered significant at an FDR corrected q-value < 0.05 (red line). Figure S4. A plot for providing evidence to select quiescent cells. Ki67 is a proliferation marker, which is not expressed in quiescent cells but is expressed in dividing cells. The graph below shows the ki67 level of cells at Day19 (red line), which was negative, indicative of lack of cell division. Figure S5. A hierarchically clustered heat map with PSI levels of significant AS events. Groups are annotated by colors in the top of row, and types of AS exons are annotated by colors in the left column. Figure S6. Skipping of CD46 exon 7 skipping in the HIV-infected T-cells. (A) Skipping of exon 7 in transcript 2 and retention of exon 8 (i.e. mutually exclusive exon event) affects the entire STP motif. (B) Lo [file 12920_2020_680_MOESM1_ESM.pdf]

Additional Files

The landscape of alternative splicing in HIV-1 infected CD4 T-cells

Seyoun Byun[1,†], Seonggyun Han[1,†], Yue Zheng[2], Vicente Planelles[2] and Younghee Lee[1,3,*]

[1]*Department of Biomedical Informatics, University of Utah School of Medicine, Salt Lake City, Utah, USA*

[2]*Department of Pathology, University of Utah School of Medicine, Salt Lake City, Utah, USA*

[3]*Huntsman Cancer Institute, University of Utah School of Medicine, Salt Lake City, Utah, USA*

[†] Contributed equally

Corresponding Authors:

Corresponding Authors: Younghee Lee, Ph.D.

Corresponding authors' address: Younghee Lee
Department of Biomedical Informatics
University of Utah School of Medicine
Salt Lake City, Utah, USA

Corresponding authors' e-mail address: younghee.lee@utah.edu

Running title

Alternative splicing in CD4 T-cells harboring HIV-1

Additional Files

Additional file 1 (.pdf):

Figure S1. Overview of AS analysis in HIV-infected and non-infected human primary resting CD4+ T-cells.

Figure S2. 23 canonical pathways enriched in 427 HIV-associated AS genes. The red line indicates the cutoff FDR value, $q < 0.05$.

Figure S3. 21 GO terms enriched in 427 HIV-associated AS genes.

Figure S4. A plot for providing evidence to select quiescent cells.

Figure S5. A hierarchically clustered heat map with PSI levels of significant AS events.

Figure S6. Skipping of *CD46* exon 7 skipping in the HIV-infected T-cells.

Additional file 2 (.xlsx): Table S1. List of AS exons in the infected T-cells

Additional file 3 (.xlsx): Table S2. List of AS genes in the enriched canonical pathways

Additional file 4 (.xlsx): Table S3. List of AS genes in the enriched GO terms

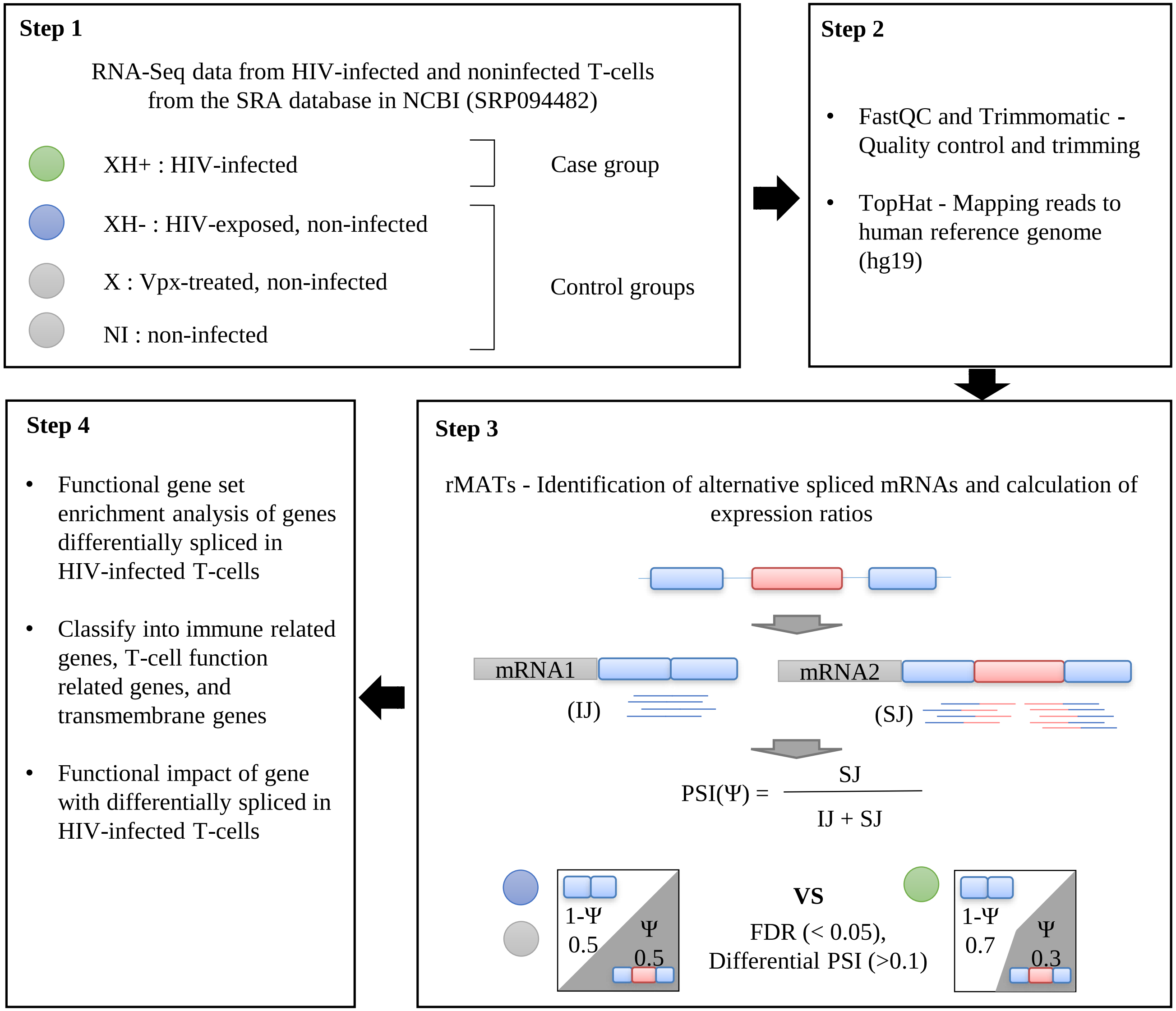

Figure S1. Overview of AS analysis in HIV-infected and non-infected human primary resting $CD4^+$ T-cells. We obtained RNA-Seq data for infected and non-infected cells from SRA (reference number SRR5071107-SRR5071122). There are four different treatment groups, and group contained four different cells . We then performed general quality control on the reads, aligned them to the hg19 human reference genome, and selected junction reads to identify alternatively spliced exons. Next, we identified mRNAs with differential alternatively splicing between the case group and each control. The percent spliced in (PSI) ratio indicates the level of differential exon expression. We adjusted q value < 0.05, and used PSI > 0.01 as the cutoff for differential splicing. Finally, we performed functional gene set enrichment analysis and classified genes with differential spliced exons in HIV infected T-cells according to their functional roles.

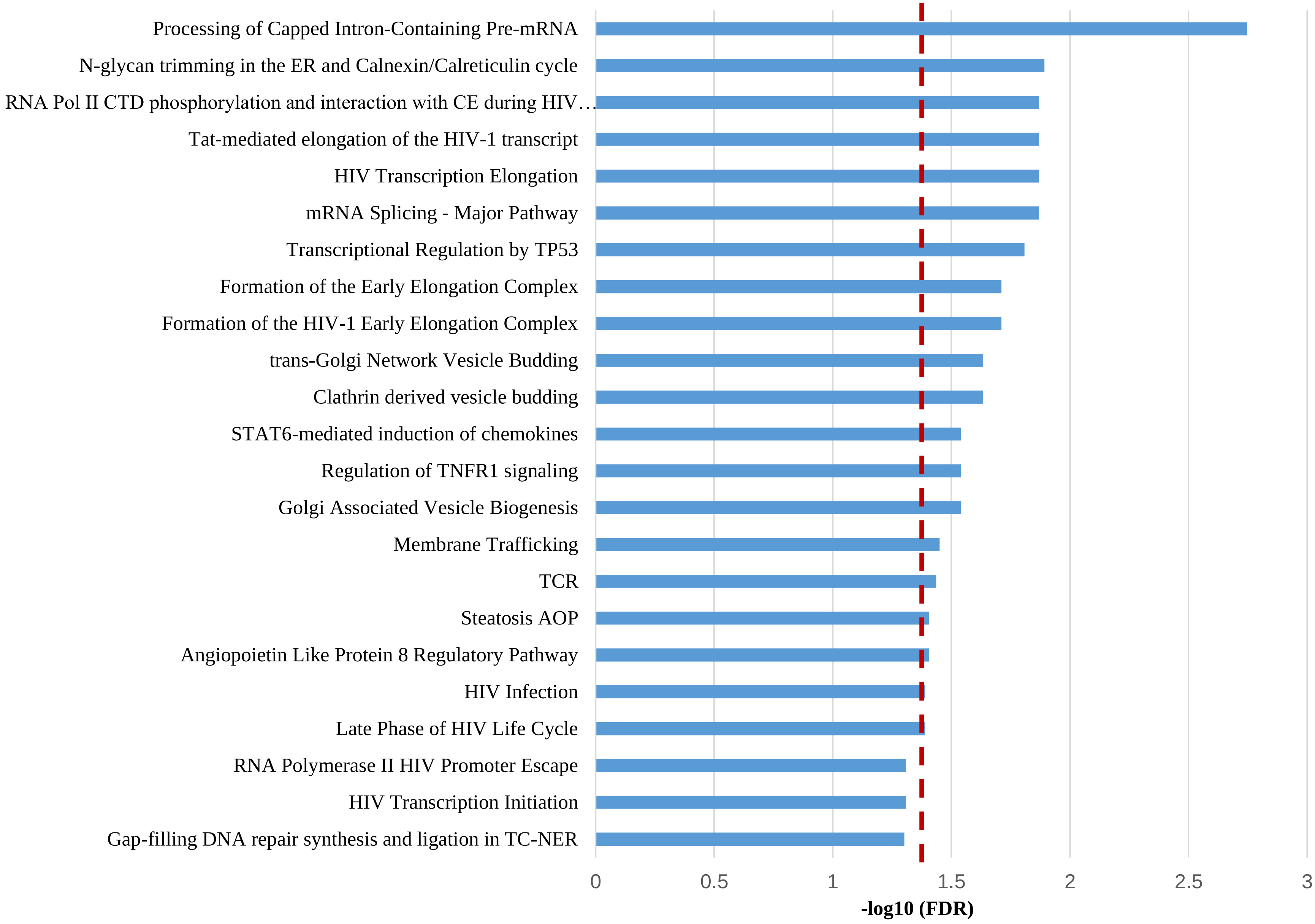

Figure S2. 23 canonical pathways enriched in 427 HIV-associated AS genes. The red line indicates the cutoff FDR value, q < 0.05.

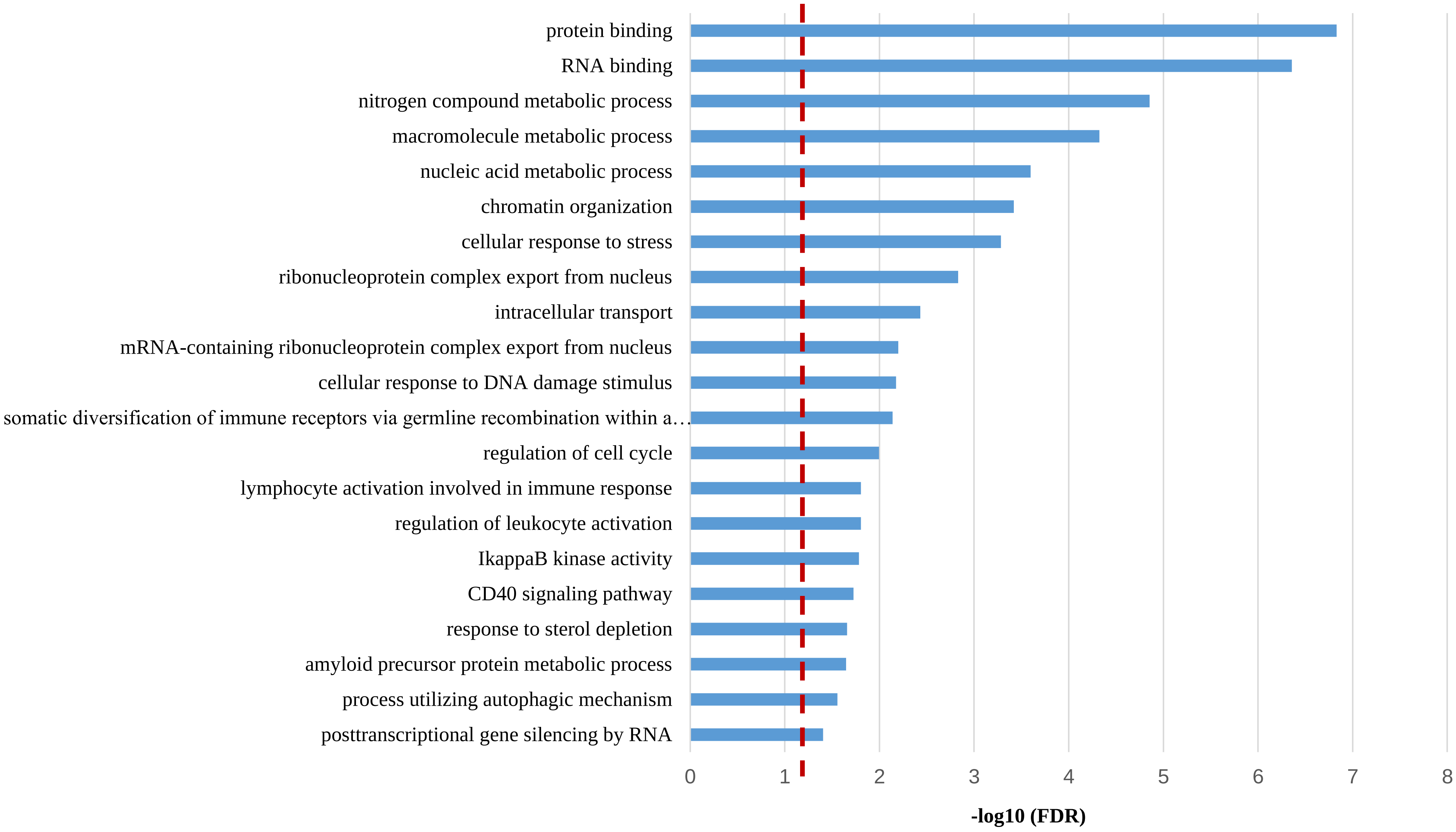

Figure S3. 21 GO terms enriched in 427 HIV-associated AS genes. GO Biological Process (BP) and Molecular Function (MF) terms were considered significant at a FDR corrected q-value < 0.05 (red line).

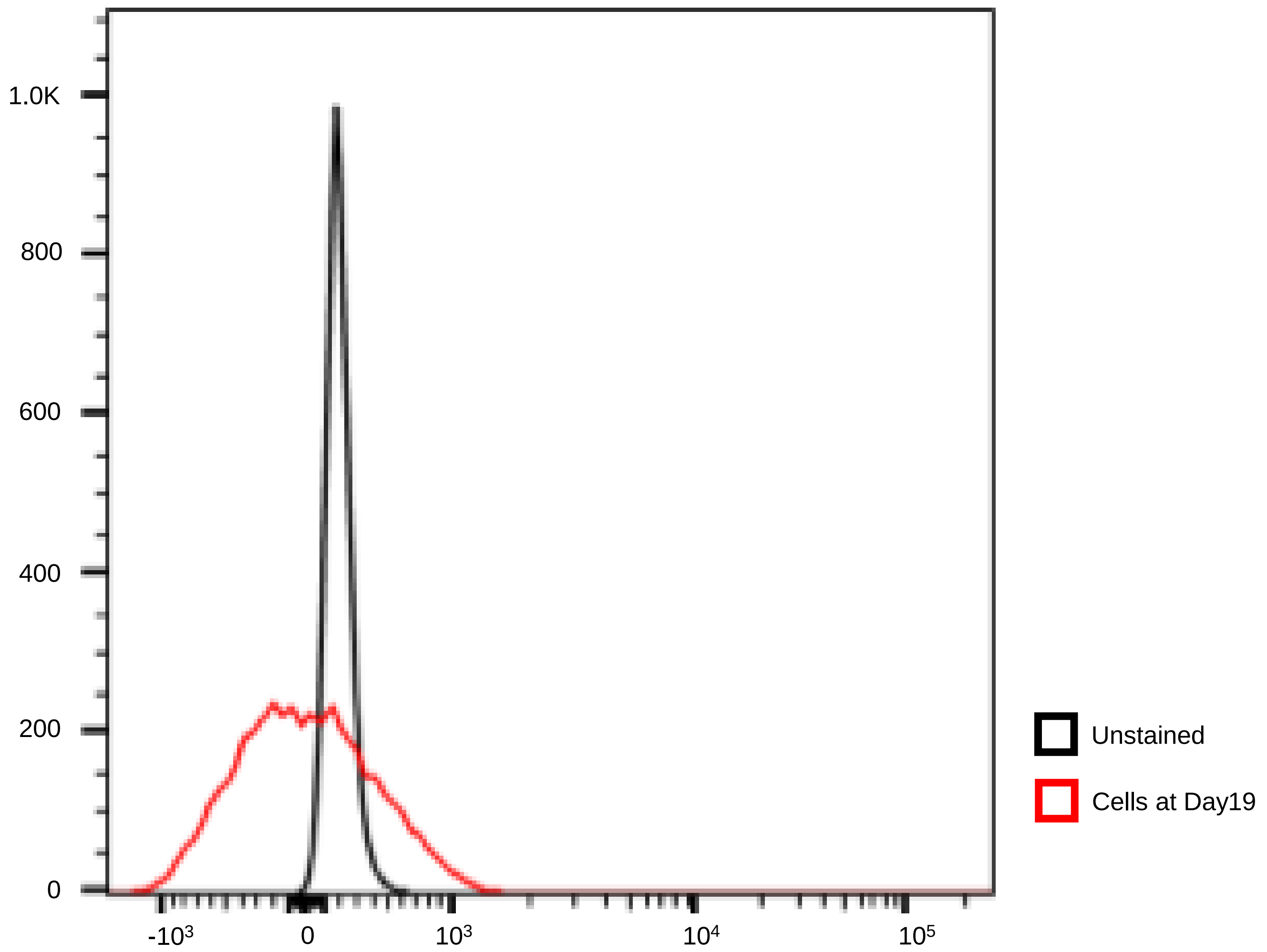

Figure S4. A plot for providing evidence to select quiescent cells. Ki67 is a proliferation marker, which is not expressed in quiescent cells but is expressed in dividing cells. The graph below shows the ki67 level of cells at Day19 (red line), which was negative, indicative of lack of cell division.

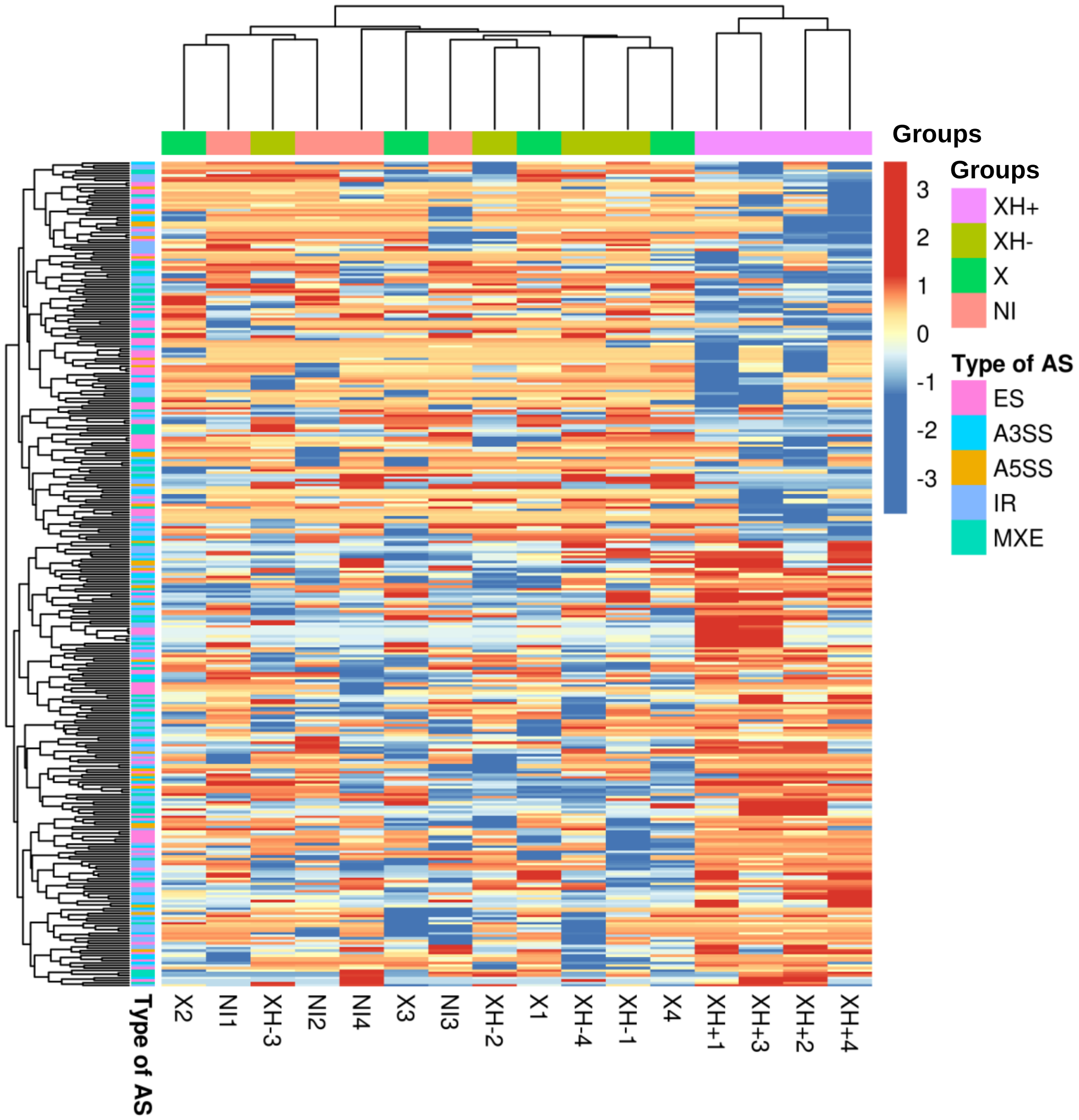

Figure S5. A hierarchically clustered heat map with PSI levels of significant AS events. Groups are annotated by colors in the top of row, and types of AS exons are annotated by colors in the left column.

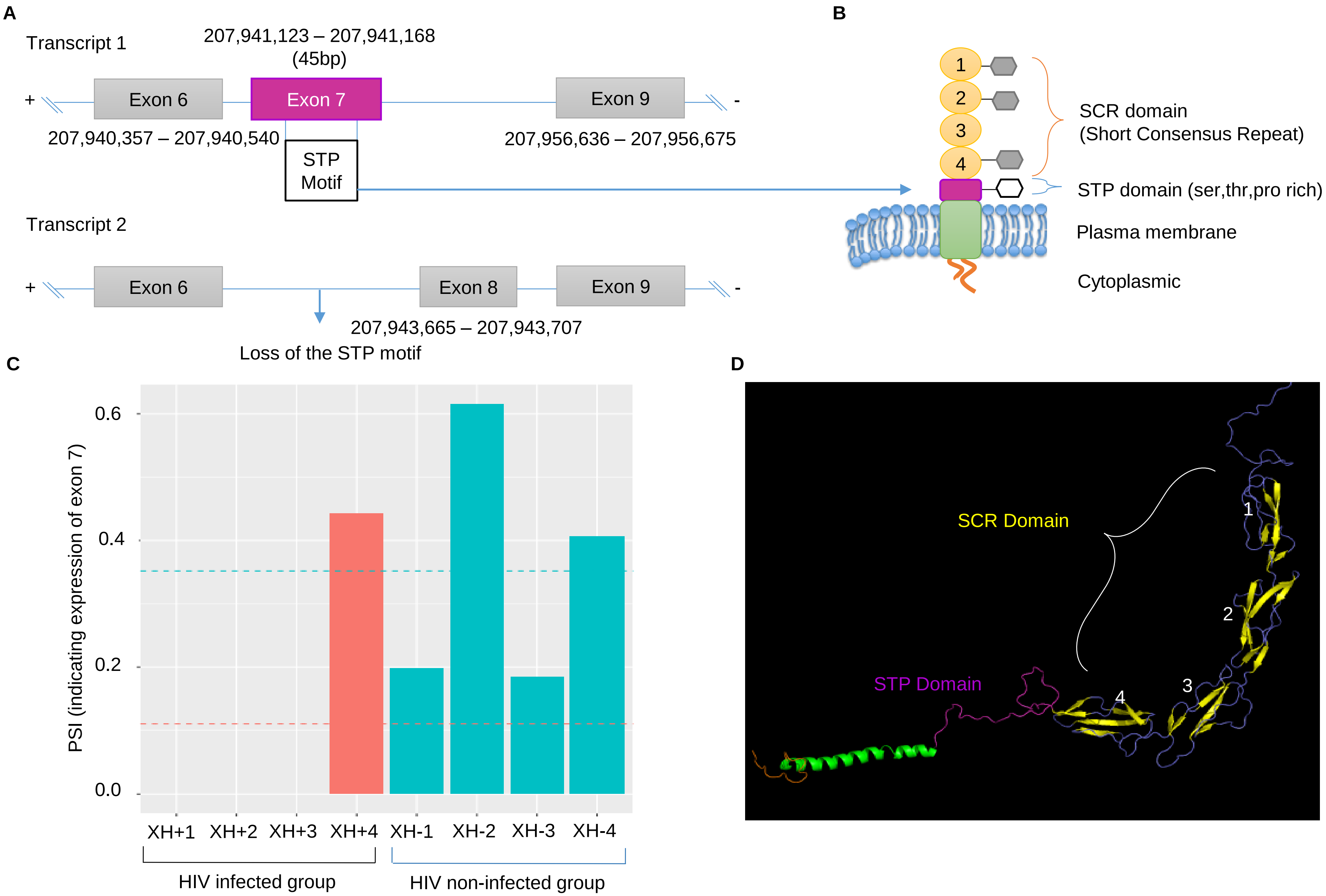

Figure S6. Skipping of CD46 exon 7 skipping in the HIV-infected T-cells. (A) Skipping of exon 7 in transcript 2 and retention of exon 8 (i.e. mutually exclusive exon event) affects the entire STP motif. (B) Location of the STP domain in CD46 protein. (C) Reduced expression of exon 7 in HIV-infected samples (p value < 7.216e-05). (D) Protein structure of CD46 with the STP domain indicated in purple.
